# Review of the fauna associated with wild and farmed mussels and oysters in the Mediterranean

**DOI:** 10.1002/brv.70193

**Published:** 2026-06-15

**Authors:** Barbara Mikac, Marina Antonia Colangelo, Alessandro Tarullo, Marco Abbiati, Eugenio Fossi, Federica Costantini

**Affiliations:** ^1^ Department of Cultural Heritage University of Bologna Via degli Ariani 1 Ravenna 48121 Italy; ^2^ Interdepartmental Centre for Industrial Research in Renewable Resources, Environment, Sea and Energy (CIRI FRAME) University of Bologna Via S. Alberto 163 Ravenna 48123 Italy; ^3^ Interdepartmental Centre for Research in Environmental Sciences (CIRSA) University of Bologna Via S. Alberto 163 Ravenna 48123 Italy; ^4^ Department of Biological, Geological and Environmental Sciences University of Bologna Via S. Alberto 163 Ravenna 48123 Italy; ^5^ National Inter‐University Consortium for Marine Sciences (CoNISMa) Piazzale Flaminio, 9 Rome 00196 Italy; ^6^ The Edge Company Via Flaminia 169 Rimini 47923 Italy; ^7^ M.A.R.E Soc. coop. a.r.l via Enrico Toti 2, 47841 Cattolica Italy

**Keywords:** biofouling, aquaculture, non‐indigenous, alien, invasive, parasite, pest, *Mytilus galloprovincialis*, *Ostrea edulis*, *Magallana gigas*

## Abstract

Mussels and oysters are important ecosystem engineers which modify the physical and chemical characteristics of the environment and create habitats that support highly diverse associated communities. In the Mediterranean Sea, the native Mediterranean mussel *Mytilus galloprovincialis* and the European flat oyster *Ostrea edulis,* together the introduced Pacific cupped oyster *Magallana gigas*, are among the most important habitat builders and farmed molluscs. The widespread disappearance of natural beds of mussels and flat oysters, driven by multiple anthropogenic disturbances, disease outbreaks, and harmful invasive species, has led to the loss of the diverse communities they support. Moreover, the proliferation of harmful invasive species is exacerbated by mollusc aquaculture, which represents one of the main vectors for the introduction and spread of non‐native species (NNS) in the Mediterranean. The aim of this review is to compile the first comprehensive database of macrofauna associated with wild and farmed *M*. *galloprovincialis*, *O*. *edulis* and *M*. *gigas* in the Mediterranean Sea, based on an extensive literature review. Specifically, we aimed to identify differences in the diversity of associated fauna among the three mollusc species, assess variability of the fauna between wild and farmed systems, highlight associated non‐native species, reveal knowledge gaps, and provide directions for future research. Overall, we recorded 782 taxa, with polychaetes, molluscs, arthropods, and ascidians representing the most species‐rich groups. Fauna associated with mussels is the most extensively studied and consequently the most diverse, with a considerably higher number of taxa reported from wild mussel habitats (647) than from mussel farms (204), likely reflecting higher environmental heterogeneity in natural contexts. By contrast, the higher diversity of fauna reported from farmed European oysters (163 taxa) compared to wild ones (50 taxa), is consistent with greater research effort focused on aquaculture systems. Fauna associated with naturalized populations of Pacific oysters (*M. gigas*) remains severely understudied, with only a few species reported to date. Information on fauna associated with molluscs is particularly lacking along much of the African coastline. Future research should therefore prioritize these understudied habitats and regions. To date, a total of 52 non‐native species have been reported from mussel and oyster habitats, with arthropods, molluscs and ascidians comprising the majority. Mussels host the highest number of NNS. Reports of NNS from mollusc habitats are increasing, particularly in mussel and oyster farms, suggesting that research efforts should focus on the detection and monitoring of NNS in Mediterranean aquaculture systems. Particular attention should be given to key groups such as non‐native ascidians and shell‐boring polychaetes, which are known pests and parasites of molluscs. This review provides a baseline for monitoring changes in communities associated with mussels and oysters in the Mediterranean. It may also serve as a reference point for the implementation of management strategies aimed at conserving and restoring mollusc habitats, as well as limiting the spread of non‐native and pest species in aquaculture systems.

## I. INTRODUCTION

Mussels and oysters are important ecosystem engineers that create habitats and modify physical and chemical characteristics of the environment (Borthagaray & Carranza, 2007; Buschbaum *et al*., 2008; Ruesink *et al*., 2005; Smyth & Roberts, 2010). Mollusc aggregations represent three‐dimensional habitats composed of densely packed shells, the interstices within the shell matrix, and sediments and biodeposits accumulated beneath the bed and among the shells. These complex habitats provide unique resources for the survival of other organisms and host highly diverse associated communities that often differ from those of the surrounding environment (Dittmann, 1990; Günther, 1996; Gutiérrez *et al*., 2003; Tsuchiya & Nishihira, 1985). In addition to these supporting services, mollusc beds offer other ecosystem services, including food provision, the regulation of natural processes such as coastal protection and nutrient removal, and cultural services such as tourism and recreational fishing (Carmichael *et al*., 2012; Isaacs, Keithly & Lavergne, 2004; La Peyre *et al*., 2015; van der Schatte *et al*., 2018). In the Mediterranean Sea, the Mediterranean mussel (*Mytilus galloprovincialis* Lamarck, 1819), the European flat oyster (*Ostrea edulis* Linnaeus, 1756) and the Pacific cupped oyster (*Magallana gigas* (Thunberg, 1793)) are among the most important habitat‐building molluscs. They also represent some of the most important farmed species in the Mediterranean (Carvalho & Guillen, 2021).

*Mytilus galloprovincialis* and *O*. *edulis* are native to the Mediterranean, Black, and Azov seas, as well as the Northeast Atlantic (Colsoul *et al*., 2021; FAO, 2022; Wenne *et al*., 2022). Both species have been introduced worldwide beyond their native range through aquaculture activities (Bromley *et al*., 2016; Colsoul *et al*., 2021; Wenne *et al*., 2022). Wild Mediterranean mussel and European flat oyster beds are subject to various anthropogenic disturbances, such as harvesting, habitat degradation, sedimentation, water pollution and global warming, which, together with disease outbreaks, have caused severe declines throughout their native distribution range (Airoldi *et al*., 2005; Rius & Zabala, 2008; Thurstan *et al*., 2024). The degradation and disappearance of wild mussel and oyster beds led to biodiversity loss, alterations in ecosystem functioning, and the deprivation of multiple ecosystem services provided by these habitats (Pogoda, 2019; Rius & Zabala, 2008; Ruesink *et al*., 2005; Thurstan *et al*., 2024; Torres *et al*., 2022). To counteract this trend, restoration measures for native oyster reefs are being planned and implemented by several European countries (Colsoul *et al*., 2021; Pogoda, 2019; Thurstan *et al*., 2024).

The Pacific oyster (*M. gigas*), native to Japan and Southeast Asia, was introduced across Europe, including the Mediterranean Sea, for aquaculture purposes in the second half of the 20th century (Grizel & Heral, 1991; Ruesink *et al*., 2005). It replaced the farming of the European flat oyster, whose production decreased due to high mortalities caused by the protozoan parasites *Marteilia refringens* Grizel, Comps, Bonami, Cousserans, Duthoit & Le Pennec, 1974 and *Bonamia ostreae* Pichot, Comps, Tigé, Grizel & Rabouin, 1980 (Buestel *et al*., 2009; Naciri‐Graven *et al*., 1998). *Magallana gigas* has also been introduced for aquaculture in many other locations worldwide and is now the most widely farmed oyster species globally (Ruesink *et al*., 2005). While initially limited to farming sites, it soon spread beyond farms, establishing wild populations. Non‐native oysters may overlap with native mussels, leading to complex competitive interactions and consequent changes in the composition of associated fauna (Kochmann *et al*., 2008). This trend is expected to intensify with global warming (Guy *et al*., 2018; King *et al*., 2021). Aquaculture is one of the main vectors for the introduction and spread of non‐native species (NNS) in Europe, with the Mediterranean Sea representing a hotspot for biological invasions (Galil *et al*., 2014; Katsanevakis *et al*., 2013; Nunes *et al*., 2014). Numerous NNS have been introduced into the Mediterranean Sea as hitchhikers of Pacific oysters imported for farming (Mineur *et al*., 2014; Ruesink *et al*., 2005). Translocation operations of molluscs are regularly performed in the Mediterranean aquaculture industry and can contribute to the transfer of NNS from one farm to another, which may subsequently spread into the surrounding environment, including natural mussel and oyster beds (Fossi *et al*., 2026). Due to their physical complexity and resource provisioning, bivalve habitats can be considered both sinks and sources for NNS. Numerous NNS are pests or parasites that can negatively influence bivalves populations and can have detrimental impacts on aquaculture (e.g. Bailey‐Brock & Ringwood, 1982; Feidantsis *et al*., 2023; Martinelli *et al*., 2020; Rodewald, Snyman & Simon, 2021; Sato‐Okoshi *et al*., 2012). Biofouling by NNS is a frequent problem in bivalve aquaculture, and the additional costs for its control can be extremely high (Bannister *et al*., 2019; Lacoste & Gaertner‐Mazouni, 2015). Apart from causing damage to bivalve production, epibionts can also alter ecological functioning of the exploited ecosystem and reduce provision of ecosystem goods and services (Mangano, Ape & Mirto, 2019; Rius, Heasman & McQuaid, 2011).

Due to the ecological and economic importance of mussel and oyster habitats in the Mediterranean, their degradation and loss, and their susceptibility to harbouring and propagating non‐native, pest, and parasite species, it is crucial to monitor the diversity of benthic communities associated with these habitats. The aim of this review is to compile the first comprehensive database of macrofauna associated with wild and farmed *M*. *galloprovincialis*, *O*. *edulis* and *M*. *gigas* in the Mediterranean Sea. We also aim to address the following questions: (*i*) are there differences in the current knowledge of the diversity of benthic macrofauna associated with the three bivalves? (*ii*) Are there differences in the current knowledge of the diversity of benthic macrofauna between farmed and wild bivalves? (*iii*) Which macrofaunal species act as pests and parasites of mussels and oysters in the Mediterranean Sea? (*iv*) Which NNS are reported in association with the three bivalves in the Mediterranean Sea, and do they differ among host species and between wild and farmed systems? (*v*) What is the temporal trend in the occurrence of NNS associated with mussels and oysters in the Mediterranean? Finally, we aim to identify gaps in current knowledge and provide directions for future research.

## II. MATERIALS AND METHODS

A systematic search of the literature was performed using Web of Science (WoS, time range 1900–2026, cut‐off date 28 February 2026). To identify publications concerning macrofauna associated with mussels and oysters in the Mediterranean, the following combination of terms was used: (Biofouling OR Fauna OR Macrofauna OR Macrobenth* OR Epibenth*) AND (Aquaculture OR Farm) AND (Mollus* OR Bivalve OR Shellfish OR Oyster* OR Mussel* OR *Mytilus galloprovincialis* OR *Crassostrea gigas* OR *Magallana gigas* OR *Ostrea edulis*) AND (Mediterranean OR Albania OR Algeria OR Bosnia and Herzegovina OR Croatia OR Cyprus OR Egypt OR France OR Greece OR Israel OR Italy OR Lebanon OR Libya OR Malta OR Monaco OR Montenegro OR Morocco OR Slovenia OR Spain OR Syria OR Tunisia OR Turkey). To focus the research on non‐native and pest species, additional terms were included: (Invasive OR Alien OR Indigenous OR Native OR Pest* OR Boring OR Parasit* OR Infest*). Additional sources listed in the references of the publications resulting from the WoS search, literature recommended by different researchers, and encountered grey literature (reports from government departments, technical and research reports, organizational annual reports, doctoral theses, dissertations, and conference proceedings) were also considered. The open ASFA database (Aquatic Sciences and Fisheries Abstracts; OpenASFA, 2025) was also explored by using, as search terms, the scientific names of mollusc species in Latin (since this is standard international taxonomic convention), and terms ‘oyster/oysters’ and ‘mussel/mussels’ in English, Italian, Spanish, Turkish, French, Croatian, Bosnian, Albanian, Slovenian, Maltese, Greek, and Arabic languages and using Latin, Cyrillic, and Arabic alphabets when appropriate: ‘*Ostrea edulis*’, ‘*Magallana gigas*’, ‘*Crassostrea gigas*’, ‘*Mytilus galloprovincialis*’, ‘oyster’, ‘oysters’, ‘ostrica’, ‘ostriche’, ‘ostra’, ‘ostras’, ‘istiridye’, ‘istiridyeler’, ‘huître’, ‘huîtres’, ‘kamenica’, ‘kamenice’, ‘ostriga’, ‘ostrige’, ‘gocë deti’, ‘goca deti’, ‘gajdra’, ‘gajdri’, ‘στρείδι’, ‘στρείδια’, 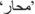, ‘mussel’, ‘cozza’, ‘cozze’, ‘mejillón’, ‘mejillones’, ‘midye’, ‘midyeler’, ‘moule’, ‘moules’, ‘dagnja’, ‘dagnje’, ‘midhe’, ‘klapavica’, ‘klapavice’, ‘maskla’, ‘maskli’, ‘μύδι’, ‘μύδια’, 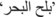, and considering years from 1930 until 2025. To better explore publications from the North African Mediterranean countries, we also performed online research of the ‘*INSTM Bulletin: Marine and Freshwater Sciences*’ (INSTM, 2025), considering the period 1924–2025 and using as the following search terms: ‘*Ostrea edulis*’, ‘*Magallana gigas*’, ‘*Crassostrea gigas*’, ‘*Mytilus galloprovincialis*’, ‘oyster’, ‘oysters’, ‘mussel’, ‘mussels’, ‘mytiliculture’, ‘ostréiculture’, ‘moule’, ‘moules’, ‘huître’, ‘huîtres’. Documents written in languages other than English were translated with Google Translate. Annual reports with overlapping data for the same combination of host bivalve species and associated species, from the same area, were considered only once [i.e. annual Institut Français de Recherche pour l'Exploitation de la Mer (IFREMER) reports on the state of Pacific oyster aquaculture along the French coasts – REMORA, reporting polychaete annelid *Polydora* as an oyster pest in the Thau Lagoon, Mediterranean Sea]. Publications were included in analyses only after critical assessment. The Mediterranean Sea was considered including the Dardanelle and Bosporus straits and the Sea of Marmara, while the Black Sea was not included. Finally, 166 publications were considered for the analyses (Table S1).

The selected literature was analysed and assigned to the following categories: bivalve host (*Mytilus*, *Ostrea* and *Magallana*), environment (Farm and Wild), pest or parasite species and non‐native species (Table S1). Information on the geographic coordinates of research locations were extracted directly from the publications or, when not reported, were approximated from Google Maps on the bases of the location description. A distribution map of the studies on macrofauna associated with the three bivalves was created on the bases of these geographic coordinates, using the R software (Becker *et al*., 2023).

Macrofaunal taxa (considering genus‐ and species‐ level records) associated with farmed and wild molluscs were extracted from the publications and compiled in the taxa table (Table S2). Organisms were considered as associated only if they were reported on or in the molluscs themselves and not merely attached to farming structures such as ropes or cages or to other surfaces in the vicinity of mussels and oysters. Meiofaunal organisms and protozoans were not considered. When appropriate, taxa were attributed to NNS (consulting the most recent lists of NNS in the Mediterranean Sea by Zenetos *et al*. (2022) and Galanidi *et al*. (2023), as well as other relevant literature where suitable) and to pest/parasite categories (based on information reported in the analysed publications). Although the amphipods *Jassa slatteryi* Conlan, 1990 and *J*. *marmorata* Holmes, 1905 are not listed as non‐native in Galanidi *et al*. (2023), they are considered NNS herein, based on the study of Conlan, Desiderato & Beermann (2021) who assigned *J*. *marmorata* as likely native to the Atlantic coasts of Canada and the USA, and *J*. *slatteryi* as native to the northeastern Pacific.

A detailed analysis of the very extensive literature concerning particular parasite species of mussels and oysters, such as the copepod crustaceans *Mytilicola intestinalis* Steuer, 1902 and *M*. *orientalis* Mori, 1935, various flatworms (Platyhelminthes) such as *Urastoma cyprinae* (Graff, 1882) and representatives of the class Trematoda [*Proctoeces maculatus* (Looss, 1901) Odhner, 1911 and *Parvatrema duboisi* (Dollfus, 1923) Bartoli, 1974], and the hydrozoan cnidarian *Eugymnanthea inquilina* Palombi, 1936, was beyond the scope of this paper. Therefore, only the principal papers reporting these parasite taxa in the target molluscs were assessed. The papers by Fernandez‐Gonzalez & Sanchez‐Jerez (2017) and Igić (1982) were considered as part of the literature analysis, but the species reported therein were not included in the species list because it was impossible to distinguish between those associated specifically with mussels and oysters.

## III. RESULTS AND DISCUSSION

### (1) State of the art of research on macrofauna associated with mussels and oysters in the Mediterranean

We have analysed 166 publications addressing macrofauna associated with mussels and oysters in the Mediterranean. In general, the fauna associated with mussels is the most studied, with most of the research focused on wild mussels concentrated in the Adriatic Sea (particularly in its northern part), followed by the Turkish and Greek coasts of the Aegean Sea, the southern French coast, and the Tyrrhenian Sea (Figs 1, 2, Table S1). Farmed mussels were best studied in the Adriatic Sea (particularly in its northern part), followed by the Ionian Sea (particularly the Gulf of Taranto) and the Aegean Sea (Greece) (Fig. 2, Table S1).

**Fig. 1:**
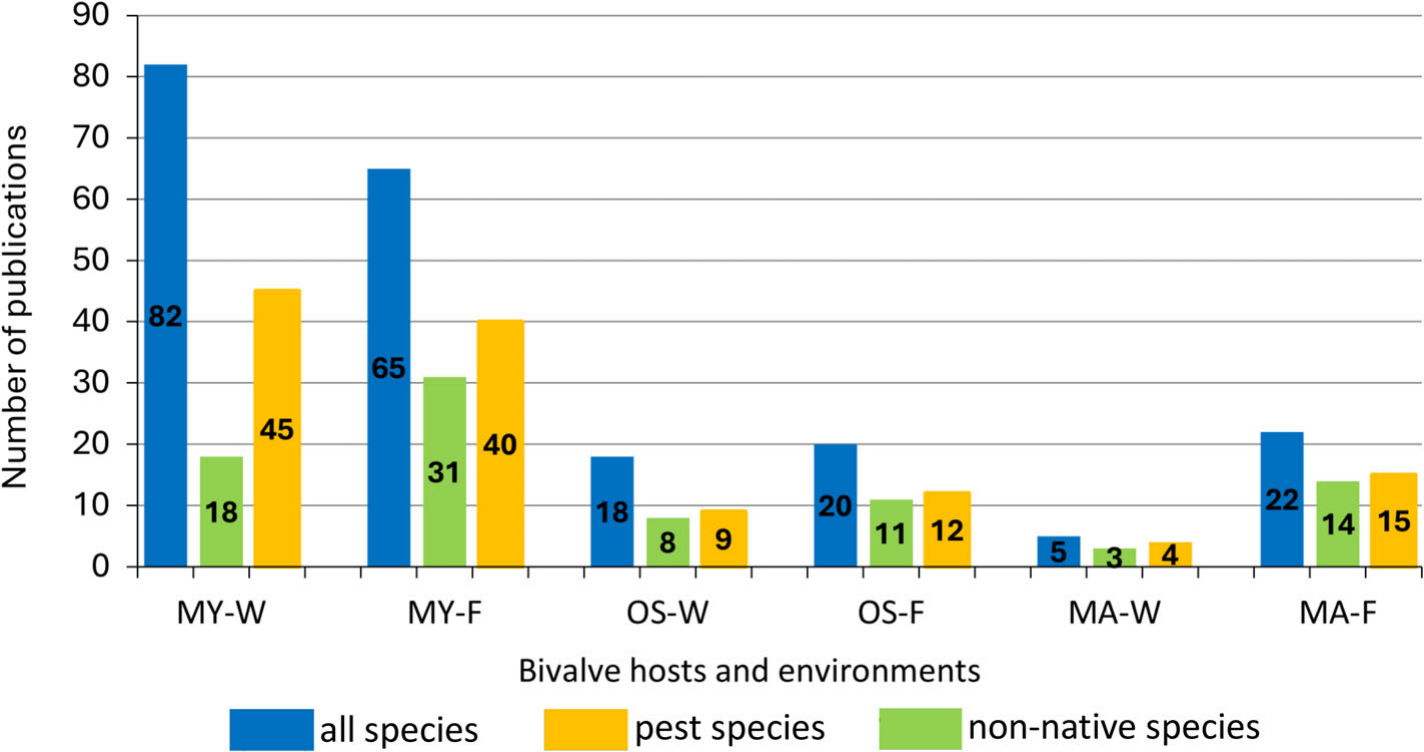
The number of publications reporting macrofaunal species associated with *Mytilus galloprovincialis* (MY), *Ostrea edulis* (OS) and *Magallana gigas* (MA) in wild (W) and farmed (F) environments in the Mediterranean Sea.

**Fig. 2:**
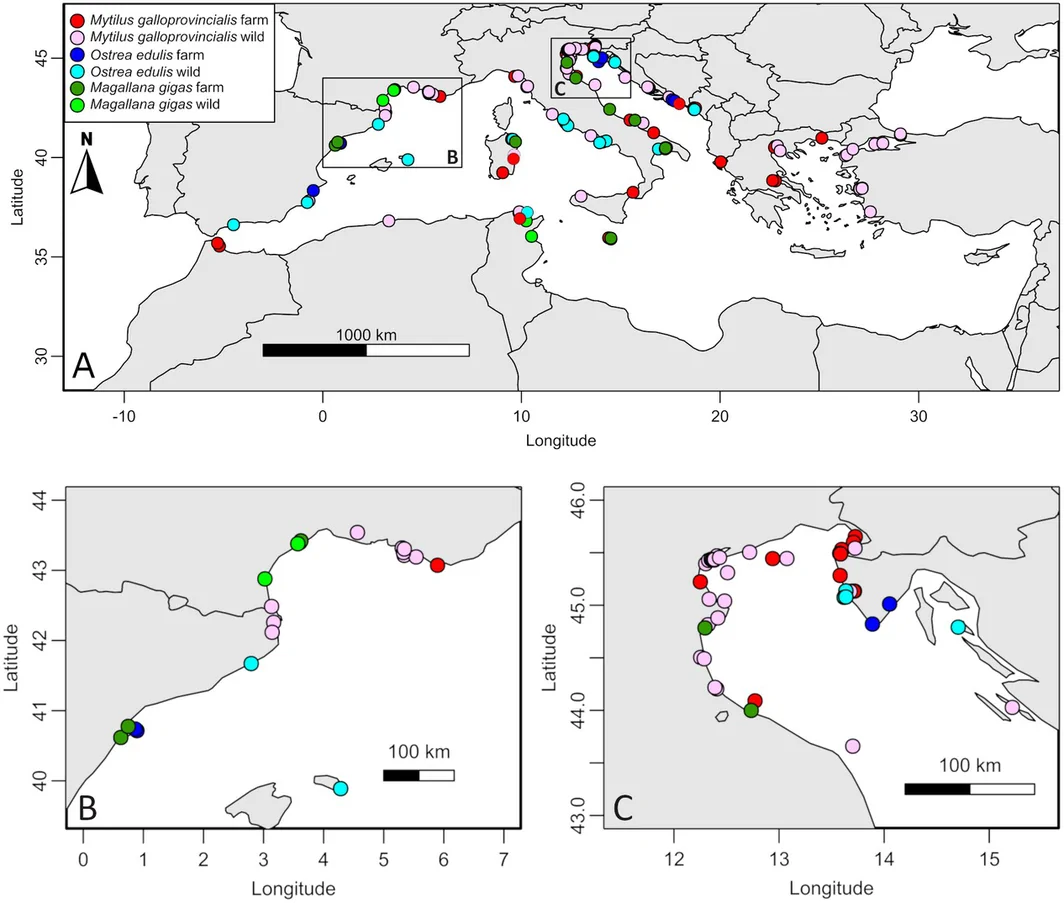
Map showing locations of studies reporting macrofauna associated with *Mytilus galloprovincialis*, *Ostrea edulis* and *Magallana gigas* in the Mediterranean Sea (A). Section (B) shows southern French and south‐eastern Spanish coasts; section (C) shows northern Adriatic coasts.

By contrast, fauna associated with European oysters in the Mediterranean has been less studied (Fig. 1). Natural flat oysters were investigated mostly in the northeastern Adriatic (Croatia), followed by the Balearic Sea (Spain), Malta, and the Tyrrhenian Sea (Fig. 2, Table S1). Studies on flat oyster farms were mostly concentrated along the eastern Adriatic coast of Croatia, where oyster farming is an important aquaculture industry (Stagličić *et al*., 2020), followed by the Ionian Sea (Taranto) and the Spanish Mediterranean coast (Alicante and the Balearic Sea).

Pacific oysters have received very limited research attention in the Mediterranean. Despite their production playing a significant role in the aquaculture industry of some of the Mediterranean countries, such as France and Italy, and continuing to develop in the basin (Graham *et al*., 2020; Mineur *et al*., 2014), research on the fauna associated with farmed Pacific oysters remains quite limited (Fig. 1). In fact, most studies are concentrated in the southern French lagoons, particularly the Thau Lagoon, and in Italy, along the western coast of the Adriatic Sea and in the Tyrrhenian Sea in Sardinia (Fig. 2). Although naturalized populations of Pacific oysters can be found throughout the Mediterranean basin (Hansen, Dolmer & Vismann, 2023), the fauna associated with this species is severely understudied (Fig. 1), with only few papers identified. Of these, Gammoudi & Tekaya (2017) reported a flatworm species in wild Pacific oysters along the Tunisian coast; Mikac *et al*. (2023, 2025) studied polychaete pests of the genus *Polydora* in farmed and wild oysters in the northern Adriatic Sea in Italy; Radashevsky *et al*. (2023a) reported *Polydora hoplura* Claparède, 1868 from the Gulf of Lion in France; and Kleitou *et al*. (2025) reported a new non‐native bryozoan attached to the shells of wild Pacific oysters in front of Koper bay in Slovenia, in the northern Adriatic Sea (Table S1).

In general, studies are concentrated mostly along the European Mediterranean coast, where also the majority of molluscs farms are located (Carvalho & Guillen, 2021) (Fig. 2, Table S1). Along the Mediterranean Asian coast, only wild mussel beds were studied, specifically in Turkey (e.g. Çinar *et al*., 2020, 2008; Kocataş, 1978). Although mussel farming is an important industry in that country, contributing about 4% of the total mussel production in the Mediterranean (Carvalho & Guillen, 2021), fauna associated with mussel farms has not been studied there. Very limited data from the African coast are available from Tunisia, Algeria and Morocco (Fig. 2, Table S1). In Tunisia, fauna associated with mussel farms has received particular attention in the Bizerte Lagoon (Fezzani *et al*., 2001; Gammoudi *et al*., 2017; Gargouri Ben Abdallah *et al*., 2012).

Comprehensive studies documenting all macrobenthic species associated with bivalves remain scarce (Agius, Schembri & Jaccarini, 1977; Antoniadou, Sarantidis & Chintiroglou, 2010; Bellan, 1969; Chintiroglou *et al*., 2004; Çinar *et al*., 2020, 2008; Curini Galletti & Galleni, 1981; D'Anna, Giaccone & Riggio, 1985; Dridi, 2000; Emrić, 1996; Fezzani *et al*., 2001; Gargouri Ben Abdallah *et al*., 2012; Igić, 1975, 1982, 1984; Kocataş, 1978; Perera, Ballesteros & Turon, 1990; Sala & Lucchetti, 2008; Topaloğlu & Kihara, 1993; Tsuchiya & Bellan‐Santini, 1989) (Table S1). Several studies describe the diversity of particular macrobenthic groups such as polychaetes (Damianidis & Chintiroglou, 2000), molluscs (Aslan‐Cihangir & Ovalis, 2013), crustaceans (Doğan, Bakir & Katağan, 2015; Kalkan, Karhan & Mutlu, 2006; Lantzouni, Voultsiadou & Chintiroglou, 1998) and flatworms (Gammoudi *et al*., 2017) (Table S1). Most of the remaining studies report single or very few taxa, which are, in many cases, of particular interest because they are pests or parasites of wild or farmed molluscs (e.g. Becker & Türkay, 2010; Canestri Trotti *et al*., 1998; Carazzi, 1893; Cerruti, 1941; Davis & Davis, 2009; Duault & Gillet, 2001; Feis *et al*., 2019; Galleni *et al*., 1980; Igić, 1972; Mikac *et al*., 2021, 2023, 2025; Prioli, 2013; Spiga, Fenzi & Salati, 2007; Yılmaz *et al*., 2020) (Table S1).

### (2) Macrofauna associated with mussels and oysters in the Mediterranean

Annelids (mostly polychaetes), molluscs, arthropods and chordates (mostly ascidians) are the richest phyla associated with mussels and oysters, comprising overall between 67% (for wild Pacific oysters) and 98% (for farmed Pacific oysters) of the total associated taxa reported until now in the Mediterranean (Fig. 3). Overall, 647 macrofaunal taxa (of which 547 are identified at species level) from 12 phyla have been reported to date in the association with wild *M*. *galloprovincialis*, whereas only 204 taxa (of which 181 are identified at species level) from 10 phyla have been reported from farmed mussels (Table S2, Fig. 3). This discrepancy is partly due to the larger number of studies on mussels in natural environments, as previously discussed (Fig. 1). It also reflects the greater environmental variability of wild mussel habitats, such as a wide range of depths (from intertidal zone to about 28 m depth), bottom types (from natural to different artificial substrates such as concrete quays and piers, wooden piles, concrete blocks, and gas platforms), frequent co‐occurrence with other habitat‐forming organisms (e.g. coralline algae, green and brown algae, sabellarid reefs) that host rich associated fauna and may further enhance local biodiversity, and the presence of different types of human disturbance (Antoniadou *et al*., 2010; Ardizzone *et al*., 1996; Bernarello *et al*., 2017; Casoli *et al*., 2019; Ceccherelli & Rossi, 1984; Cerrano *et al*., 2015; Çinar *et al*., 2020; D'Anna *et al*., 1985; Emrić, 1996; Marchini, Gauzer & Occhipinti‐Ambrogi, 2004; Marzialetti, Nicoletti & Ardizzone, 2009; Tsuchiya & Bellan‐Santini, 1989). By contrast, mussel farms share similar environmental characteristics that support a more limited macrofaunal diversity. Moreover, repetitive disturbances caused by farming operations and the ephemeral nature of farmed mussel clumps hinder the development of a highly diverse associated fauna. Only 119 taxa are shared between wild and farmed mussels.

**Fig. 3:**
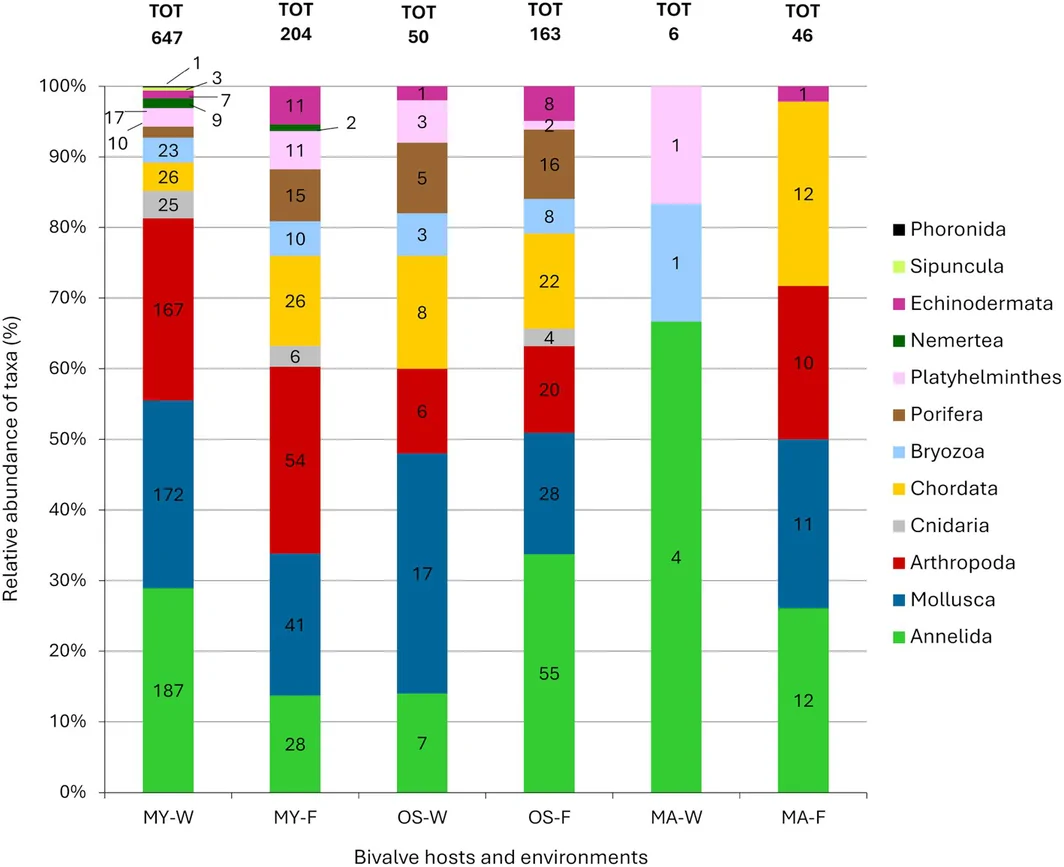
Relative abundance and number of taxa (reported within each coloured bar) per phylum of macrofauna associated with *Mytilus galloprovincialis* (MY), *Ostrea edulis* (OS) and *Magallana gigas* (MA) in wild (W) and farmed (F) environments in the Mediterranean Sea. Total number of taxa is reported above each column.

The fauna associated with farmed European oysters has been the target of several comprehensive studies, particularly in the Adriatic Sea (Agius *et al*., 1977; Igić, 1975, 1982, 1984; Parenzan, 1961; Perera *et al*., 1990; Stjepčević, 1973; Zavodnik & Igić, 1968), whereas interest in wild *O*. *edulis* has been more limited (Igić, 1975, 1982, 1984). This greater research effort resulted in more than a threefold higher number of taxa reported from European oyster farms compared with wild oysters (163 taxa, of which 138 are identified at species level, from 9 phyla in farmed oysters, compared with 50 taxa of which 43 are identified at species level, from 8 phyla in wild oysters) (Table S2, Figs 1, 3).

Forty‐six taxa (of which 41 are identified at species level) from 5 phyla have been reported in association with farmed Pacific oysters in the Mediterranean Sea (Table S2, Fig. 3). By contrast, only six species have been reported from wild Pacific oysters: four non‐native spionid polychaetes, *Polydora websteri* Hartman in Loosanoff & Engle, 1943, *Polydora caeca* Blake & Kudenov, 1978, *P*. *hoplura* and *P*. *cornuta* Bosc, 1902, non‐native bryozoan *Watersipora subatra* (Ortmann, 1890) and the flatworm *Discocelis tigrina* (Blanchard, 1847) (Table S2). Conversely, interest in wild and farmed Pacific oysters outside the Mediterranean Sea has been much greater, with studies reporting the rich benthic fauna associated with this species (e.g. Arakawa, 1990; Guy *et al*., 2018; Rocha, 2009; Rodriguez & Ibarra‐Obando, 2008). A comparison of epibiota on wild populations of non‐native *M*. *gigas* and native *O*. *edulis* along the northern European coast revealed significant differences between the epibiotic communities of the two species, with epibiont species richness being significantly lower on *M*. *gigas* (Guy *et al*., 2018). Unfortunately, the lack of data in the Mediterranean prevents similar comparisons within the basin. The Pacific oyster is a highly adaptable species and, with global warming, it is likely to expand its populations in the Mediterranean, potentially overrunning mussels habitats and the already depleted native oyster populations, as already observed in northern Europe (Guy *et al*., 2018; Kochmann *et al*., 2008). This expansion could consequently lead to shifts in oyster epibiota diversity. The issue is particularly relevant considering the numerous oyster‐reef restoration projects in Europe and the Mediterranean, since a further spread of *M*. *gigas* might interfere with successful reef restoration. Therefore, research on the fauna associated with Pacific oysters in the Mediterranean Sea should be encouraged. Such studies would enable future evaluations of the impact of the spread of this species on benthic diversity in the basin.

### (3) Non‐native species (NNS) associated with mussels and oysters in the Mediterranean

We recorded 52 non‐native species (NNS) from 7 phyla reported in association with mussels and oysters in the Mediterranean (35 from farmed and 38 from wild environments), with arthropods being the richest phylum (13 species) (Table 1). The highest number of NNS, 35 and 26, respectively, were reported from wild and farmed Mediterranean mussels (Table 1, Fig. 4A). It is evident that NNS richness is correlated with the number of publications dealing with NNS associated with different molluscs (Figs 1, 4). Several species listed in the paper by Di Blasio *et al*. (2023), who reviewed non‐native species associated with commercial bivalves in Italian marine waters, were not considered here because they were found in the environment surrounding mussels and oysters, but not on the bivalves themselves.

**Table 1:** Non‐native species (NNS) associated with wild (‐W) and farmed (‐F) *Mytilus galloprovincialis* (MY), *Ostrea edulis* (OS) and *Magallana gigas* (MA) in the Mediterranean. Year = year of the first report in the Mediterranean. Country = Country of first report in the Mediterranean. Origin = native range of the species. Vectors of introduction: S = ship transport; A = aquaculture; C = corridors/canals. † = pest or parasite species. Literature used for NNS information: Conlan *et al.* (2021); Davis & Davis (2008); de Barros, da Rocha & Pie (2009); Di Blasio *et al.* (2023); Dyrynda *et al.* (2000); Galanidi *et al.* (2023); Gauff, *et al.* (2023a,b); Kerckhof (2002); Langeneck *et al.* (2020); Malan *et al.* (2020); Mikac *et al.* (2025); Ordóñez *et al.* (2015); Pérez‐Portela *et al.* (2013); Radashevsky *et al.* (2023a,b); Ragkousis *et al.* (2023); Ruffo (1959); Sato‐Okoshi *et al.* (2023); Schembri (1979); WoRMS (2025); Zenetos *et al.* (2010, 2017, 2022); Zenetos & Galanidi (2020).

Species	MY‐W	MY‐F	OS‐W	OS‐F	MA‐W	MA‐F	Year	Country	Origin	Vector
**Annelida**										
Polychaeta										
†*Dipolydora giardi* (Mesnil, 1893)	‐	‐	X	‐	‐	‐	1981	Algeria	NE Pacific	A
*Ficopomatus enigmaticus* (Fauvel, 1923)	‐	X	‐	‐	‐	‐	1919	Italy	Indian	S
*Hydroides dirampha* Mörch, 1863	‐	X	‐	‐	‐	‐	1868	Italy	NW Atlantic	S
*Hydroides elegans* (Haswell, 1883)	X	X	‐	X	‐	‐	1868	Italy	Indo Pacific	S
*Polydora cornuta* Bosc, 1802	X	‐	‐	‐	X	X	1986	Turkey	W Atlantic	SA
†*Polydora caeca* Webster, 1879	‐	‐	‐	X	X	X	2023	Italy	SW Pacific	A
†*Polydora hoplura* Claparède, 1868	X	‐	X	X	X	X	1868	Italy	NW Pacific	S
†*Polydora websteri* Hartman in Loosanoff & Engle, 1943	X	‐	‐	‐	X	X	2023	Italy	NW Pacific	A
*Pseudonereis anomala* Gravier, 1899	X	‐	‐	‐	‐	‐	1933	Egypt	Indian	SC
*Pseudopolydora paucibranchiata* (Okuda, 1937)	X	‐	‐	‐	‐	‐	1977	Italy	NW Pacific	SA
*Streblospio gynobranchiata* Rice & Levin, 1998	X	‐	‐	‐	‐	‐	2002	Turkey	W Atlantic	S
*Syllis pectinans* Haswell, 1920	X	‐	‐	‐	‐	‐	1975	Spain	W Pacific	S
**Arthropoda**										
Hexanauplia										
Sessilia										
*Amphibalanus eburneus* (Gould, 1841)	X	X	X	X	‐	‐	1750–1800	Italy	W Atlantic	SA
*Balanus trigonus* Darwin, 1854	X	X	X	X	‐	‐	1927	Italy	Indo Pacific	S
*Megabalanus tintinnabulum* (Linnaeus, 1758)	‐	X	‐	‐	‐	‐	1791	Italy	Indo Pacific	S
Cyclopoidia										
†*Myicola ostreae* Hoshina & Sugiura, 1953	‐	‐	‐	‐	‐	X	1972	France	NW Pacific	A
†*Mytilicola orientalis* Mori, 1935	X	X	‐	‐	‐	X	1979	France	NW Pacific	A
Malacostraca										
Amphipoda										
*Caprella scaura* Templeton, 1836	X	X	‐	‐	‐	‐	1994	Italy	W Indian	S
*Jassa marmorata* Holmes, 1905	X	X	‐	‐	‐	‐	1890	Italy	NW Atlantic	S
*Jassa slatteryi* Conlan, 1990	X	‐	‐	‐	‐	‐	⁓1952	Italy	NE Pacific	S
*Stenothoe georgiana* Bynum & Fox, 1977	X	‐	‐	‐	‐	‐	2010	Spain	W Atlantic	S
Isopoda										
*Paracerceis sculpta* Holmes, 1904	‐	X	‐	‐	‐	‐	1978	Tunisia	NE Pacific	S
*Paradella dianae* (Menzies, 1962)	X	X	‐	‐	‐	‐	1985	Italy	NE Pacific	S
Decapoda										
†*Callinectes sapidus* Rathbun, 1896	‐	‐	‐	‐	‐	X	1947	Greece	W Atlantic	S
*Dyspanopeus sayi* (Smith, 1869)	‐	X	‐	‐	‐	‐	1985	Tunisia	NW Atlantic	S
**Bryozoa**										
Gymnolaemata										
*Celleporaria brunnea* (Hincks, 1884)	X	‐	‐	‐	‐	‐	2003	Lebanon	NE Pacific	S
*Tricellaria inopinata* d'Hondt & Occhipinti Ambrogi, 1985	X	X	‐	‐	‐	‐	1982	Italy	Indo Pacific	A
*Watersipora subatra* (Ortmann, 1890)	‐	‐	‐	‐	X	‐	2019	France	?Pacific	S
**Chordata**										
Ascidiacea										
†*Aplidium* cf. *accarense* (Millar, 1953)	‐	X	‐	‐	‐	‐	2012	Spain	NE Atlantic	S
*Ascidiella aspersa* (Müller, 1776)	X	X	X	X	‐	X	1828	Italy	NW Atlantic	S
†*Ciona robusta* Hoshino & Tokioka, 1967	X	X	X	X	‐	X	1816	Egypt	NW Pacific	S
†*Clavelina oblonga* (Herdman, 1880)	‐	X	‐	‐	‐	X	1928	Italy	NW Atlantic	A
†*Didemnum vexillum* Kott, 2002	X	‐	‐	‐	‐	X	2007	Italy	NW Pacific	A
†*Diplosoma listerianum* (Milne Edwards, 1841)	X	X	X	X	‐	‐	1877	Italy	NE Atlantic	S
*Microcosmus exasperatus* Heller, 1878	X	‐	‐		‐	‐	1998	Tunisia	Indo Pacific	S
*Microcosmus squamiger* Michaelsen, 1927	‐	‐	‐	X	‐	‐	1962	Tunisia	SW Pacific	S
*Polyandrocarpa zorritensis* (Van Name, 1931)	‐	‐	‐	X	‐	‐	1974	Italy	SE Pacific	A
*Styela clava* Herdman, 1882	‐	X	‐	‐	‐	X	2005	France	NW Pacific	A
†*Styela plicata* (Lesueur, 1823)	X	X	‐	X	‐	X	1877	Italy	NW Pacific	S
**Cnidaria**										
Anthozoa										
*Diadumene cincta* Stephenson, 1925	X	‐	‐	‐	‐	‐	1993	Italy	NE Atlantic	S
**Mollusca**										
Bivalvia										
*Anadara transversa* (Say, 1822)	X	X	‐	‐	‐	X	1972	Turkey	NW Atlantic	S
*Arcuatula senhousia* (Benson, 1842)	X	X	‐	‐	‐	‐	1960	Israel	NW Pacific	C
*Brachidontes pharaonis* (P. Fischer, 1870)	X	‐	‐	‐	‐	‐	1876	Egypt	Indian/Red Sea	C
*Magallana gigas* (Thunberg, 1793)	X	‐	‐	‐	‐	‐	1964	Italy	NW Pacific	A
†*Pinctada radiata* (Leach, 1814)	X	X	X	‐	‐	‐	1874	Egypt	Indo Pacific	C
*Rapana venosa* (Valenciennes, 1846)	X	‐	‐	‐	‐	‐	1973	Italy	NW Pacific	A
*Ruditapes philippinarum* (A. Adams & Reeve, 1850)	X	‐	‐	‐	‐	‐	1980	France	NW Pacific	A
*Steromphala cineraria* (Linnaeus, 1758)	‐	X	‐	‐	‐	‐	1976	Malta	NE Atlantic	A
*Xenostrobus securis* (Lamarck, 1819)	X	‐	‐	‐	‐	‐	1991	Italy	SW Pacific	A
Gastropoda										
*Crepidula fornicata* (Linnaeus, 1758)	‐	X	X	‐	‐	‐	1957	France	NW Atlantic	A
*Polycera hedgpethi* Er. Marcus, 1964	X						1986	Italy	NE Atlantic	S
**Porifera**										
Calcarea										
†*Paraleucilla magna* Klautau, Monteiro & Borojevic, 2004	X	X	‐	‐	‐	‐	2000	Spain	SW Atlantic	S
**TOTAL**	**35**	**26**	**9**	**11**	**5**	**14**				

**Fig. 4:**
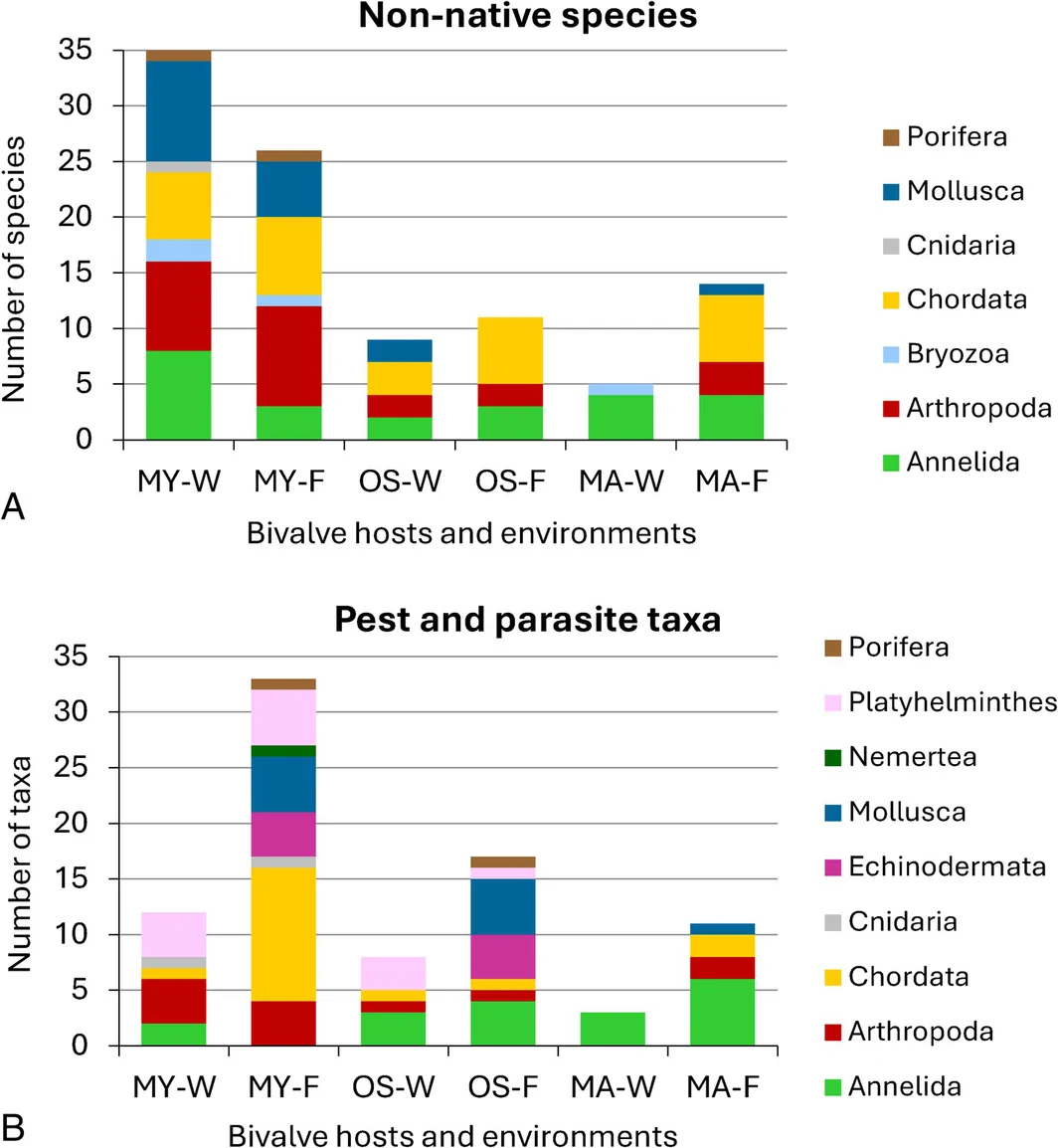
Number of non‐native species (A) and pest and parasite taxa (B) per phyla, reported from wild (‐W) and farmed (‐F) *Mytilus galloprovincialis* (MY), *Ostrea edulis* (OS) and *Magallana gigas* (MA) in the Mediterranean Sea.

Overall, most NNS both on both wild and farmed mussels and oysters belong to arthropods and ascidians, whereas in wild mussels non‐native molluscs are also well represented (Fig. 4A). Several non‐native polychaetes are present in wild mussels and Pacific oysters. Our results reflect the general pattern of NNS in the Mediterranean Sea, where arthropods are the second‐richest group after molluscs and have shown an accelerated rate of establishment in recent years (Galanidi *et al*., 2023; Zenetos *et al*., 2022). Among arthropods, non‐native barnacles (i.e. *Amphibalanus eburneus* (Gould, 1841); *Balanus trigonus* Darwin, 1854; and *Megabalanus tintinnabulum* (Linnaeus, 1758)) and amphipods (*Caprella scaura* Templeton, 1836; *Jassa marmorata* Holmes, 1905; *Jassa slatteryi* Conlan, 1990; and *Stenothoe georgiana* Bynum & Fox, 1977) are particularly well represented among both wild and farmed mussels. On the other hand, non‐native cyclopoids *Myicola ostreae* Hoshina & Sugiura, 1953 and *Mytilicola orientalis* are notable parasites of oysters and mussels, respectively.

Ascidians represent one of the most common groups of invaders in the marine environment, and their rate of introduction has increased in recent decades, mainly due to the shipping and aquaculture activities (Dupont *et al*., 2010; López‐Legentil, Turon & Planes, 2006; Ordóñez *et al*., 2015, 2016; Raijman Nagar & Shenkar, 2016; Turon, Nishikawa & Rius, 2007). Non‐native ascidians are well known to cause significant damage to aquaculture facilities (Clarke & Therriault, 2007; Daigle & Herbinger, 2009; Feidantsis *et al*., 2023; Papadopoulos *et al*., 2023). In fact, among 11 non‐native ascidians listed herein, 6 are mollusc pests (Table 2). Congruently, the first NNS reported in association with farmed and wild mussels and farmed Pacific oysters in the Mediterranean were ascidians. For mussels, Parenzan (1961) first reported the ascidians *Ciona robusta* Hoshino & Tokioka, 1967 and *Styela plicata* (Lesueur, 1823) encumbering farmed mussels in Taranto Bay (Italy), whereas Bellan‐Santini (1969) recorded *Diplosoma listerianum* (Milne Edwards, 1841) from an infralittoral wild mussel bed in the Bay of Marseille (France). Since then, reports of new NNS associated with both farmed and wild mussels have steadily increased up to the present day (Fig. 5A,B). The report of the non‐native ascidian *Microcosmus exasperatus* Heller, 1878 from an oyster farm at the Ebro river delta by Perera *et al*. (1990) was herein considered to refer to *Microcosmus squamiger* Michaelsen, 1927. This is based on conclusions of Turon *et al*. (2007) and Ramos‐Espla, Izquierdo & Çinar (2013) who consider *M*. *squamiger* to be distributed in the western Mediterranean basin, whereas *M*. *exasperatus* should be restricted to the eastern part. Carazzi (1893) was the first to report the non‐native polychaete pest *Polydora hoplura* boring into wild and farmed flat oysters along the coast of Naples (Italy). Non‐native species were reported again in association with flat oysters only after several decades. In particular, Parenzan (1961, 1967) found *P*. *hoplura* in farmed flat oysters, whereas Igić (1972) found the ascidian *D*. *listerianum* on wild oysters in the Northern Adriatic Sea. Very few new NNS were reported from wild and farmed flat oysters from the 1970s to the late 1990s (Fig. 5C, D). Only recently have new non‐native polychaete pests of the genus *Polydora* been reported from farmed and wild *O*. *edulis* (Mikac *et al*., 2025; Radashevsky *et al*., 2023a,b). Following the rationale of Mikac *et al*. (2025), the species reported as *Polydora coeca* Webster, 1879 from wild mussel beds in Izmir bay (Turkey) by Çinar *et al*. (2008), was herein considered *Dipolydora coeca* (Örsted, 1843), not *Polydora caeca* Webster, 1879. Çinar *et al*. (2008) did not provide any detail of worm morphology. Moreover, in the Checklist of Annelida from the coasts of Turkey, Çinar, Dağlı & Kurt Şahin (2014) included only *Dipolydora coeca* (Örsted, 1843), whereas *P*. *coeca* Webster was not mentioned in that or any subsequent publication. The first NNS recorded in Pacific oyster farms in the Mediterranean was again the ascidian *C*. *robusta*, found by Korringa & Lambert (1951) in the Thau Lagoon (France), where it completely covered farmed oysters. From then until the early 2000s, only a few NNS were newly reported from Pacific oyster farms (Fig. 5F). However, since 2000, reports of new NNS in association with farmed *M*. *gigas* have steadily increased, confirming that this expanding Mediterranean industry plays a role in the transfer of NNS (Carvalho & Guillen, 2021; Di Blasio *et al*., 2023). Recent research involving Adriatic oyster farmers revealed numerous translocations of oyster adults and juveniles between Pacific oyster farms inside and outside the Mediterranean Sea, which presumably contribute to the introduction and spread of NNS (Fossi *et al*., 2026). Whereas most NNS in European oyster farms were reported from 1970–1990, in Pacific oyster farms NNS have been recorded principally since 2000. This trend is consistent with large‐scale replacement of European oyster farming with Pacific oysters starting in the 1970s, following high mortalities of the native *O*. *edulis* caused by protozoan diseases bonamiosis and marteiliosis (Buestel *et al*., 2009; Naciri‐Graven *et al*., 1998). Only in 2023 were non‐native species reported for the first time in association with wild Pacific oysters (Fig. 5E). In particular, Mikac *et al*. (2023) and Radashevsky *et al*. (2023a) found *Polydora* pests boring in farmed Pacific oysters in the Northern Adriatic Sea (Croatia) and the Gulf of Lion (France), respectively. Among the only five publications documenting fauna associated with wild Pacific oysters, four report non‐native species (Kleitou *et al*., 2025; Mikac *et al*., 2023, 2025; Radashevsky *et al*., 2023a). It is likely that further research will reveal additional NNS associated with Pacific oysters.

**Table 2:** Pest and parasite taxa associated with wild (‐W) and farmed (‐F) *Mytilus galloprovincialis* (MY), *Ostrea edulis* (OS) and *Magallana gigas* (MA) in the Mediterranean Sea. The symbol (X) is used only when a taxon is reported as a pest or parasite of a given farmed or wild mollusc in the cited literature. Literature = publications reporting the pest or parasite relationship. † = non‐native species.

Species	MY‐W	MY‐F	OS‐W	OS‐F	MA‐W	MA‐F	Literature
**Annelida**							
Polychaeta							
†*Dipolydora giardi* (Mesnil, 1893)	‐	‐	X	‐	‐	‐	Radashevsky *et al*. (2023b)
*Polydora ciliata* (Johnston, 1838)	X	‐	X	X	‐	X	Çaglar (1954); Carazzi (1893); Parenzan (1961, 1967); Spiga *et al*. (2007)
†*Polydora caeca* Webster, 1879	‐	‐	‐	X	X	X	Mikac *et al*. (2025)
†*Polydora hoplura* Claparède, 1868	‐	‐	X	X	X	X	Carazzi (1893); Labura & Hrs‐Brenko (1990a); Nel *et al*. (1996); Parenzan (1961, 1967); Presečki‐Labura (1987); Radashevsky *et al*. (2023a)
*Polydora* sp.	‐	‐	‐	X	‐	X	Duault & Gillet (2001); Fleury *et al*. (1998); Fossi *et al*. (2026); Gavrilović *et al*. (2008); Lukić (2011); Prioli (2013)
†*Polydora websteri* Hartman in Loosanoff & Engle, 1943	‐	‐	‐	‐	X	X	Mikac *et al*. (2025)
**Arthropoda**							
Hexanauplia							
Cyclopoidia							
†*Myicola ostreae* Hoshina & Sugiura, 1953	‐	‐	‐	‐	‐	X	Pollio (1981)
*Mytilicola intestinalis* Steuer, 1902	X	X	‐	‐	‐	‐	Canestri Trotti *et al*. (1998); Da Ros & Massignan (1985); Feis *et al*. (2019); Hrs‐Brenko (1964); Karagiannis *et al*. (2013); Presečki‐Labura (1987); Rayyan, Christidis & Chintiroglou (2002); Rayyan, Photis & Chintiroglou (2004); Steuer (1902); Yılmaz *et al*. (2020)
†*Mytilicola orientalis* Mori, 1935	X	X	‐	‐	‐	X	Feis *et al*. (2019); Kovačić *et al*. (2017); Pollio (1981); Yılmaz *et al*. (2020)
*Pseudomyicola spinosus spinosus* (Raffaele & Monticelli, 1885)	X	‐	‐	‐	‐	‐	Laubier & Reyss (1964); Stock (1993)
Sessilia							
*Amphibalanus amphitrite* (Darwin, 1854)	‐	X	‐	X	‐	‐	Cerruti (1926); Parenzan (1961); Stjepčević (1973)
*Perforatus perforatus* (Bruguière, 1789)	‐	X	‐	‐	‐	‐	Parenzan (1961)
Malacostraca							
Decapoda							
†*Callinectes sapidus* Rathbun, 1896	‐	‐	‐	‐	‐	X	Fossi *et al*., 2026
*Pinnotheres pisum* (Linnaeus, 1767)	X	‐	X	‐	‐	‐	Becker & Türkay (2010); Berner (1952); Lauckner (1983)
**Chordata**							
Ascidiacea							
†*Aplidium* cf. *accarense* (Millar, 1953)	‐	X	‐	‐	‐	‐	Melki *et al*. (2025)
*Botrylloides leachii* (Savigny, 1816)	‐	X	‐	‐	‐	‐	Santacroce *et al*. (2008)
*Botryllus* sp.	‐	X	‐	‐	‐	‐	Feidantsis *et al*. (2023); Papadopoulos *et al*. (2023)
*Botryllus schlosseri* (Pallas, 1766)	‐	X	‐	‐	‐	‐	Parenzan (1961); Santacroce *et al*. (2008)
†*Ciona robusta* Hoshino & Tokioka, 1967	‐	X	‐	‐	‐	‐	Feidantsis *et al*. (2023); Papadopoulos *et al*. (2023); Parenzan (1961); Santacroce *et al*. (2008)
*Clavelina lepadiformis* (Müller, 1776)	‐	X	‐	‐	‐	‐	Parenzan (1961); Santacroce *et al*. (2008)
†*Clavelina oblonga* (Herdman, 1880)	‐	X	‐	‐	‐	X	Feidantsis *et al*. (2023); Fossi *et al*. (2026); Majnarić *et al*. (2022); Ordóñez *et al*. (2016); Papadopoulos *et al*. (2023); Privileggio *et al*. (2024); Topić Popović *et al*. (2025)
*Didemnum candidum* Savigny, 1816	‐	X	‐	‐	‐	‐	Parenzan (1961)
*Didemnum maculosum* (Milne Edwards, 1841)	‐	X	‐	‐	‐	‐	Parenzan (1961)
†*Didemnum vexillum* Kott, 2002	‐	‐	‐	‐	‐	X	Casso *et al*. (2018)
*Didemnum* sp.	‐	X	‐	‐	‐	‐	Santacroce *et al*. (2008)
†*Diplosoma listerianum* (Milne Edwards, 1841)	X	X	X	X	‐	‐	Igić (1972)
†*Styela plicata* (Lesueur, 1823)	‐	X	‐	‐	‐	‐	Feidantsis *et al*. (2023); Papadopoulos *et al*. (2023); Parenzan (1961); Santacroce *et al*. (2008)
**Cnidaria**							
Hydrozoa							
*Eugymnanthea inquilina* Palombi, 1936	X	X	‐	‐	‐	‐	Bommarito *et al*. (2022); Karagiannis *et al*. (2013); Lauckner (1983); Mladineo *et al*. (2012); Parenzan (1961); Piraino *et al*. (1994); Presečki‐Labura (1987); Rayyan, Photis & Chintiroglou (2004); Žižek, Gombač & Pogačnik (2012)
**Echinodermata**							
Asteroidea							
*Astropecten aranciacus* (Linnaeus, 1758)	‐	X	‐	X	‐	‐	Stjepčević (1973)
*Astropecten spinulosus* (Philippi, 1837)	‐	X	‐	X	‐	‐	Stjepčević (1973)
*Coscinasterias tenuispina* (Lamarck, 1816)	‐	X	‐	X	‐	‐	Stjepčević (1973)
*Marthasterias glacialis* (Linnaeus, 1758)	‐	X	‐	X	‐	‐	Stjepčević (1973)
**Mollusca**							
Bivalvia							
*Anomia* sp.	‐	X	‐	‐	‐	‐	Fossi *et al*. (2026)
*Hiatella arctica* (Linnaeus, 1767)	‐	‐	‐	X	‐	‐	Lukić (2011)
†*Pinctada radiata* (Leach, 1814)	‐	X	‐	‐	‐	‐	Theodorou, Rizos & Tzovenis (2008)
*Rocellaria dubia* (Pennant, 1777)	‐	‐	‐	X	‐	X	Mikac *et al*. (2021); Morton, Peharda & Petrić, 2011; Parenzan (1961)
Gastropoda							
*Bolinus brandaris* (Linnaeus, 1758)	‐	X	‐	X	‐	‐	Stjepčević (1973)
*Hexaplex trunculus* (Linnaeus, 1758)	‐	X	‐	X	‐	‐	Stjepčević (1973)
*Ocenebra erinaceus* (Linnaeus, 1758)	‐	X	‐	X	‐	‐	Stjepčević (1973)
**Nemertea**							
Hoplonemertea							
*Emplectonema gracile* (Johnston, 1837)	‐	X	‐	‐	‐	‐	Parenzan (1961, 1967)
**Platyhelminthes**							
Trematoda							
*Gymnophallus minutus* (Cobbold, 1859)	‐	X	‐	‐	‐	‐	Parenzan (1961)
*Gymnophallus perligena* Palombi, 1940	‐	X	‐	‐	‐	‐	Parenzan (1961)
*Parvatrema duboisi* (Dollfus, 1923) Bartoli, 1974	X	‐	‐	‐	‐	‐	Bommarito *et al*. (2022); Dollfus (1923); Lauckner (1983); Yılmaz *et al*. (2020)
*Parvatrema* sp.	X	‐	‐	‐	‐	‐	Marchiori *et al*. (2023); Puljas & Burazin (2022)
*Postmonorchis* sp.	‐	‐	X	‐	‐	‐	Mancini *et al*. (2018)
*Prosorhynchoides haimeana* (Lacaze‐Duthiers, 1854)	‐	‐	X	‐	‐	‐	Lacaze‐Duthiers (1854); Lauckner (1983)
*Prosorhynchoides* sp.	‐	‐	X	‐	‐	‐	Mancini *et al*. (2018)
*Proctoeces maculatus* (Looss, 1901) Odhner, 1911	X	X	‐	‐	‐	‐	Da Ros & Massignan (1985); Dubois (1907); Gargouri Ben Abdallah *et al*. (2012); Karagiannis *et al*. (2013); Lauckner (1983); Rayyan, Photis & Chintiroglou (2004)
Fecampiida							
*Urastoma cyprinae* (Graff, 1882)	X	X	‐	‐	‐	‐	Bommarito *et al*. (2022); Canestri Trotti *et al*. (1998); Karagiannis *et al*. (2013); Mladineo *et al*. (2012); Rayyan, Photis & Chintiroglou (2004); Yılmaz *et al*. (2020)
Polycaldida							
*Discocelis tigrina* (Blanchard 1847)	‐	‐	‐	X	‐	‐	Cerruti (1926)
*Stylochus mediterraneus* (Galleni, 1976)	X	X	‐	‐	‐	‐	Fossi *et al*. (2026); Galleni *et al*. (1980); Gammoudi *et al*. (2017); Privileggio *et al*. (2024)
**Porifera**							
Calcarea							
†*Paraleucilla magna* Klautau, Monteiro & Borojevic, 2004	‐	X	‐	‐	‐	‐	Longo, Mastrototaro & Corriero, 2007; Longo *et al*. (2012)
Demospongiae							
*Cliona* sp.	‐	‐	‐	X	‐	‐	Lukić (2011)
**TOTAL**	**12**	**33**	**8**	**17**	**3**	**11**	

**Fig. 5:**
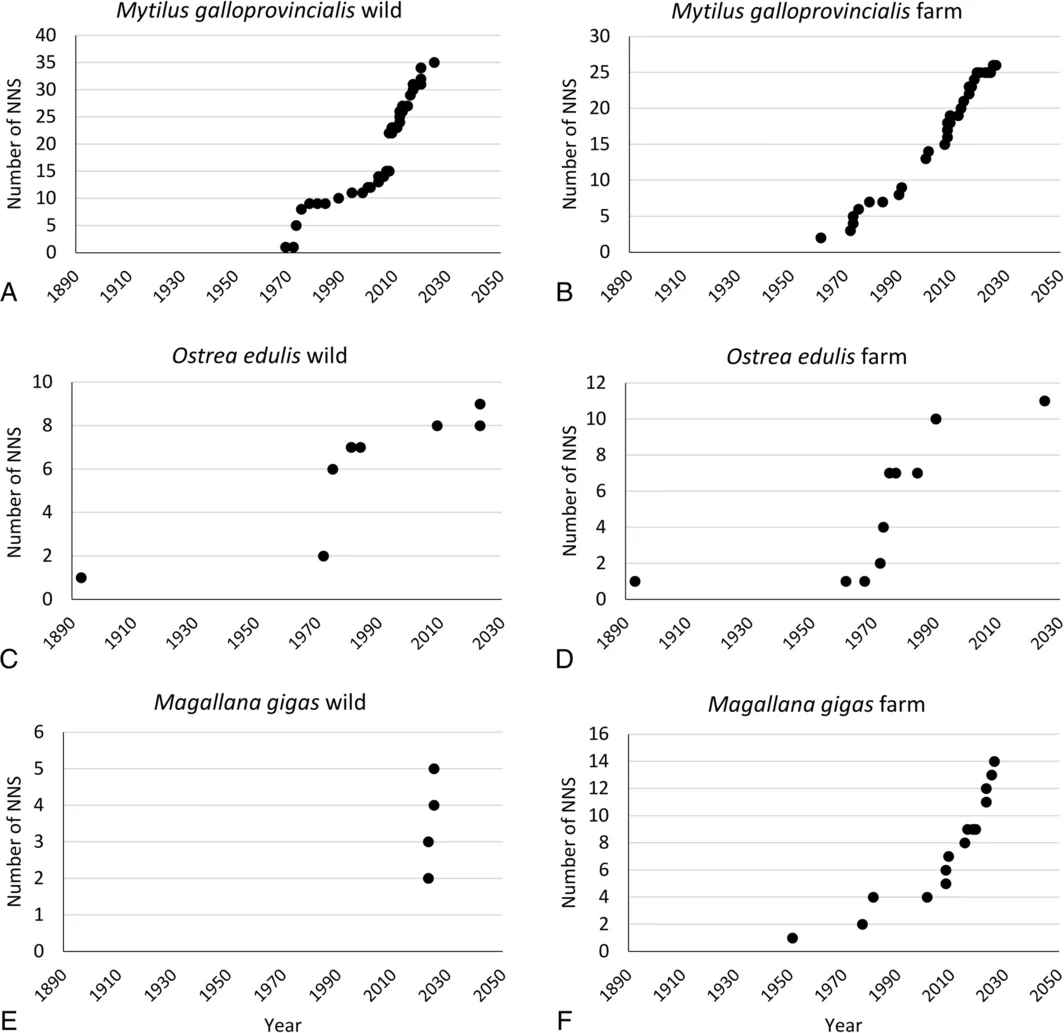
Species accumulation curves for new records of non‐native species found in association with farmed and wild mussels and oysters in the Mediterranean Sea.

Most of the NNS are of western Atlantic and western Pacific origin, and most were introduced into the Mediterranean Sea by shipping, followed by aquaculture (Table 1). The majority have been recorded in association with bivalves in hotspot areas for the NNS introduction in the Mediterranean Sea, such as ports (e.g. Thessaloniki Bay in the Aegean Sea: Antoniadou *et al*., 2010; Chintiroglou *et al*., 2004; Damianidis & Chintiroglou, 2000; Gerovasileiou *et al*., 2017; Izmir Bay in Turkey: Çinar *et al*., 2008; Doğan, 2016; Doğan *et al*., 2015; Kocataş, 1978), lagoons (e.g. northern Adriatic Italian lagoons: Lazzari & Rinaldi, 1994; Marchini *et al*., 2004; Russo, 2001; Thau Lagoon in France: Davis & Davis, 2008, 2009; Korringa & Lambert, 1951; Mazouni, Gaertner & Deslous‐Paoli, 2001; Pollio, 1981; Bizerte Lagoon in Tunisia: Dridi, 2000), and aquaculture sites (e.g. Thau Lagoon in France; Bizerte Lagoon in Tunisia; Mar Piccolo in the Gulf of Taranto in Italy: Katsanevakis *et al*., 2014; Longo, Mastrototaro & Corriero, 2007; Parenzan, 1961; Pierri, Longo & Giangrande, 2010; Lake Butrint in Albania: Osca & Crocetta, 2020).

### (4) Pests and parasites of mussels and oysters in the Mediterranean

Of the 53 pests and parasites affecting mussels and oysters, the majority are flatworms and ascidians (Table 2). In general, more pests and parasites are reported from farmed molluscs, presumably reflecting the greater research interest in these species within aquaculture because of their negative impact on production. Fifteen of these pest and parasite species are non‐native (Fig. 4B).

The most frequently reported pests and parasites are polydorid polychaetes (*Polydora ciliata* (Johnston, 1838), *P*. *hoplura* and *Polydora* sp.), copepod crustaceans (*M*. *intestinalis* and *M*. *orientalis*), trematode flatworms (*P*. *maculatus* and *U*. *cyprinae*), ascidians (*C*. *robusta*, *S*. *plicata*, *Clavelina oblonga* (Herdman, 1880)) and the hydrozoan *E*. *inquilina* (Table S2). Members of the *Polydora*‐group are well‐known borers in mussels and oysters, whose infestation can severely damage shells and compromise hosts health and performance (Chambon *et al*., 2007; Fossi *et al*., 2026; Handley & Bergquist, 1997; Spiga *et al*., 2007). Numerous *Polydora* pests are NNS in different parts of the world, where they are transported by infested oysters through aquaculture and have caused high oyster mortalities and severe negative effects on both oyster farming and natural oyster beds (Malan *et al*., 2020; Martinelli *et al*., 2020, 2024; Mikac *et al*., 2025; Ogburn, White & McPhee, 2007; Rodewald *et al*., 2021; Sato‐Okoshi *et al*., 2012). Due to their cryptic nature, introduced *Polydora* species can be erroneously identified and remain unnoticed in the new environment (Malan *et al*., 2020; Mikac *et al*., 2025). Recently, new Mediterranean invaders, *Polydora websteri* and *P*. *caeca*, were reported for the first time boring in farmed and wild Pacific oysters and wild mussels in the Northern Adriatic Sea (Mikac *et al*., 2023, 2025). Considering recent findings of *P*. *websteri* in different locations along European coasts, including the Mediterranean Sea, and the numerous transfers of molluscs between aquaculture facilities within the Mediterranean basin and with European farms outside the basin, which may contribute to the further spread of this species, it is probable that *P*. *websteri* has a much wider distribution in the Mediterranean (Fossi *et al*., 2026; Mikac *et al*., 2025). Further research efforts should be undertaken to reveal the presence of this species in the Mediterranean, particularly in oyster farms. Unidentified *Polydora* sp. have often been reported as infesting farmed oysters in the Mediterranean (Table 2). Moreover, some studies reported shell‐boring *Polydora ciliata* and *P*. *hoplura*, the taxonomy and distribution of which have recently been questioned and reviewed in different parts of the world (Table 2; Radashevsky, Choi & Gambi, 2017; Radashevsky & Pankova, 2006; Sato‐Okoshi *et al*., 2017). Some of these previous records may belong to other non‐native cryptic species; thus, further efforts to study *Polydora* borers in molluscs should be made to disentangle the identity of existing species and to promptly detect the presence of new NNS in the Mediterranean. Studies based on integrative approach combining morphological and molecular analyses for species identification are particularly important for the early detection of these invasive species (Malan *et al*., 2020; Martinelli *et al*., 2020; Mikac *et al*., 2025; Rodewald *et al*., 2021; Sato‐Okoshi *et al*., 2017).

Copepods are noteworthy parasites of molluscs. *Mytilicola intestinalis* is a parasite in mussels' intestines, known to damage the intestinal epithelium and to cause catastrophic mortalities of mussel populations (Korringa, 1951; Lauckner, 1983). Numerous research and monitoring programmes have been carried out to understand biology and ecology of this species in the Mediterranean, and several management measures have been imposed to prevent its accidental spread by aquaculture (see literature in Table 2). *Mytilicola orientalis* and *Myicola ostreae* are two non‐native copepods indigenous to the northwest Pacific Ocean (Korea and Japan), first reported in the Mediterranean in 1979 in the Thau Lagoon (France), where they were introduced with Pacific oysters imported for aquaculture (Clanzig, 1989; Pollio, 1981). *Mytilicola orientalis*, commonly called red worm, is a parasite of the intestinal tracts of bivalves and is considered one of the 100 worst invasive species in the Mediterranean Sea (Mori, 1935; Streftaris & Zenetos, 2006). On the other hand, the copepod *M*. *ostreae*, a parasite of the gills of several bivalves, particularly *M*. *gigas*, has never been found in the Mediterranean after its first report, and clear data on its exact distribution in the basin are lacking. This parasite causes gill lesions and reduces host growth rates, and it can lead to mortality when infestation is very intense (Batista *et al*., 2009; Stock, 1993). The species is established in several European countries along the Atlantic coast and the North Sea, where it spread through infested oysters. Thus, it is plausible that it is more widely distributed also in the Mediterranean, and further research in the basin should be conducted to clarify its presence, particularly in areas where *M*. *gigas* has been translocated and established.

Trematode flatworms are considered the most significant metazoan parasites of bivalves (Lauckner, 1983). During recent research on the fauna associated with mussels in the Adriatic Sea, farmers reported an unusually high infestation by flatworms feeding on mussels, coinciding with increased mussel mortality (Fossi *et al*., 2026; Mikac, personal observation). In recent years, massive die‐offs of wild and farmed mussels in the Mediterranean have been primarily attributed to rising global temperatures, which directly affect mussels and make them more vulnerable to additional environmental stressors, including parasite attacks (Bracchetti *et al*., 2024; Salini *et al*., 2024; Vivarelli, 2024). Mussels weakened by heat waves or by infestation of other pest species, such as ascidians, are more susceptible to predation by flatworms (Privileggio *et al*., 2024). Consequently, the negative impacts of flatworms on Mediterranean mussels are expected to grow in the coming years. Further efforts should be made to reveal promptly any potential non‐native flatworm pests.

Biofouling by ascidians encumbers culture ropes and cages, negatively affecting farmed molluscs and causing major damage to the Mediterranean aquaculture industry (Feidantsis *et al*., 2023; Papadopoulos *et al*., 2023). The most problematic species are the non‐native *C*. *robusta*, *S*. *plicata* and *C*. *oblonga*, which currently have a worldwide distribution and are spreading through shipping and aquaculture (Tables 1 and 2). As shown for these three species, misidentification can obscure their non‐native status. Therefore, particular attention should be paid to their accurate identification in mollusc farms, since new invaders can easily go unnoticed and be inadvertently transported across the Mediterranean through aquaculture activities (Brunetti *et al*., 2015; Ordóñez *et al*., 2016; Pennati *et al*., 2015). In particular, the spread of the non‐native *Styela clava* Herdman, 1882, native to the northwest Pacific, and first reported in the Mediterranean in 2004 in the Thau Lagoon, should be carefully monitored, since the species might become a pest in mollusc aquaculture, as already observed outside the Mediterranean (Bourque *et al*., 2005; Davis & Davis, 2010; Kang, Kim & Yoon, 1980; LeBlanc *et al*., 2007; Nel, Coetzee & Van Niekerk, 1996).

Different treatments have been applied in the aquaculture industry to control or remove non‐native pests, often resulting in high costs, limited effectiveness and negative effects on cultured animals (Handley, 2002; Lacoste & Gaertner‐Mazouni, 2015; LeBlanc *et al*., 2007; McKindsey *et al*., 2007). Thus, avoidance and prevention should be considered the best first‐line defence for the management of these species (Bannister *et al*., 2019). In the Mediterranean, efforts should focus on adopting comprehensive measures for managing pests in mollusc aquaculture. These measures should include early detection through efficient research and monitoring in both wild and farmed environments, strong legislative actions, and collaboration among stakeholders from the scientific, aquaculture, and government sectors.

## IV. CONCLUSIONS

(1) This is the first review of the macrofauna associated with the mussel *M*. *galloprovincialis* and the oysters *O*. *edulis* and *M*. *gigas* in the Mediterranean Sea. It will serve as a baseline for monitoring changes in the biodiversity of benthic assemblages associated with these molluscs. It will also provide a reference point for implementing management measures for the conservation and restoration of mollusc habitats and for containing the spread of non‐native and pest species through mollusc aquaculture.
(2) Polychaetes, molluscs, arthropods, and ascidians are the richest groups associated with mussels and oysters. The fauna associated with mussels is the best studied and, consequently, the most diverse, with a considerably higher number of taxa reported from wild mussels than from mussel farms. This likely reflects greater environmental variability combined with higher habitat integrity in natural environments. By contrast, the lower diversity of fauna associated with wild European oysters reflects the lower research effort compared with that devoted to European oyster farms.
(3) Although naturalized populations of Pacific oysters are spread throughout the Mediterranean basin, the fauna associated with these habitats remains largely understudied. Further research should be encouraged to assess the diversity of these habitats, in order to enable the evaluation of the impacts of Pacific oyster expansion, predicted to increase with rising sea temperature, on benthic biodiversity in the Mediterranean.
(4) The majority of the 52 NNS associated with mussels and oysters belong to arthropods, molluscs, and ascidians. The number of NNS observed in different molluscs reflects the research effort dedicated to them. In fact, the highest numbers of NNS are reported in association with the best‐studied mussels. Whereas more NNS were reported from better explored wild mussel environments than from farmed ones, in oysters more NNS are known from farmed environments, where research has been more extensive.
(5) The observed growing trend in new NNS reports from molluscs, and particularly from mussel and oyster farms, indicates that dedicated research efforts should be made for the detection and monitoring of NNS in aquaculture. Farming practices often include the exchange of juvenile and adult molluscs between farms both within and outside the Mediterranean basin, which will probably contribute to the introduction of new NNS and the spread of existing ones.
(6) Various species, particularly ascidians, arthropods, and flatworms, are pests and parasites, especially in farmed mussels and oysters, causing serious damage to mollusc aquaculture. Out of 53 reported pest and parasite species, fifteen are non‐native. Further research should focus on some target pest species, such as representatives of the *Polydora*‐complex, ascidians and flatworms, which are well‐known global invaders and may go unnoticed because of their cryptic nature. Studies employing an integrative approach, including taxonomy based on morphology and DNA barcoding, will be particularly valuable for the early detection of these invaders. In the coming years, heatwaves may weaken molluscs, making them more susceptible to parasite attacks. Therefore, future research should aim to unravel these mechanisms.
(7) Studies on fauna associated with molluscs are concentrated almost exclusively along the European and, to a lesser extent, Asian coasts of the Mediterranean, whereas information is lacking for most of the extensive African coast. Future studies should focus on characterising the fauna associated with molluscs in these understudied areas.
(8) Natural mussel and oyster beds in the Mediterranean Sea are increasingly affected by various human disturbances, leading to their degradation and loss, with a consequent loss of ecosystem services they provide. Monitoring changes in the extent of mussel and oyster beds, as well as conducting comprehensive characterisation and continuous monitoring of communities associated with these habitats, should be encouraged to detect alterations in their status and to implement effective conservation and management measures.

## Supporting information


**Table S1.** Publications reporting fauna associated with farmed and wild *Mytilus galloprovincialis*, *Ostrea edulis*, and *Magallana gigas* in the Mediterranean Sea.

**Table S2.** List of taxa reported in association with farmed and wild *Mytilus galloprovincialis*, *Ostrea edulis*, and *Magallana gigas* in the Mediterranean Sea.
